# A Review of the Molecular Mechanisms Underlying Cardiac Fibrosis and Atrial Fibrillation

**DOI:** 10.3390/jcm10194430

**Published:** 2021-09-27

**Authors:** Grażyna Sygitowicz, Agata Maciejak-Jastrzębska, Dariusz Sitkiewicz

**Affiliations:** Department of Clinical Chemistry and Laboratory Diagnostics, Medical University of Warsaw, 02-097 Warsaw, Poland; agata.maciejak@wum.edu.pl (A.M.-J.); dariusz.sitkiewicz@gmail.com (D.S.)

**Keywords:** cardiac fibrosis, atrial fibrillation, neurohormonal mechanisms, MMPs, miRNAs

## Abstract

The cellular and molecular mechanism involved in the pathogenesis of atrial fibrosis are highly complex. We have reviewed the literature that covers the effectors, signal transduction and physiopathogenesis concerning extracellular matrix (ECM) dysregulation and atrial fibrosis in atrial fibrillation (AF). At the molecular level: angiotensin II, transforming growth factor-β1, inflammation, and oxidative stress are particularly important for ECM dysregulation and atrial fibrotic remodelling in AF. We conclude that the Ang-II-MAPK and TGF-β1-Smad signalling pathways play a major, central role in regulating atrial fibrotic remodelling in AF. The above signalling pathways induce the expression of genes encoding profibrotic molecules (MMP, CTGF, TGF-β1). An important mechanism is also the generation of reactive oxygen species. This pathway induced by the interaction of Ang II with the AT_2_R receptor and the activation of NADPH oxidase. Additionally, the interplay between cardiac MMPs and their endogenous tissue inhibitors of MMPs, is thought to be critical in atrial ECM metabolism and fibrosis. We also review recent evidence about the role of changes in the miRNAs expression in AF pathophysiology and their potential as therapeutic targets. Furthermore, keeping the balance between miRNA molecules exerting anti-/profibrotic effects is of key importance for the control of atrial fibrosis in AF.

## 1. Introduction

Myocardial fibrosis, causing atrial fibrillation (AF), is one of the main factors leading to heart remodelling. It should be noted, however, that the process of fibrosis causes significant changes both in the ventricles and in the atria of the heart. Ventricular fibrosis leads to the development of heart failure, while atrial fibrosis leads to the generation of atrial fibrillation episodes. Pathological phenotypes in atrial and ventricular fibrosis are different. Ventricular fibrosis is primarily induces pumping dysfunction (systolic dysfunction and diastolic dysfunction) whereas atrial fibrosis is responsible for cardiac conduction abnormalities. The process of atrial fibrosis includes various individual and multifactorial processes with underlying complicated interactions between cellular and neurohormonal mediators [1]. Myocardial remodelling is a set of complex structural and haemodynamic processes occurring both at structural (changes of heart shape and size) [2] and cellular (changes in cell composition of the cardiac tissue) as well as molecular (changes in the expression levels of cardiomyocyte proteins) levels [3,4]. Extremely important is also the effect of pathological stimuli, starting from heart damage, pressure overload, inflammatory condition or neurohormonal activation, which lead to activation of many remodelling pathways. Among these pathways, of particular importance are: apoptosis, necrosis, energy metabolism changes, oxidative stress, proliferation of fibroblasts and extracellular matrix (ECM) activation [2].

Atrial fibrillation is one of the most frequent tachyarrhythmias in the clinical practice [5,6], being a frequent cause of thromboembolic events [6]. Furthermore, AF development is closely related to patients’ age and comorbidities, although every third case of AF can develop without comorbidities [7]. The clinically recognized risk factors of AF include: arterial hypertension, congestive heart failure, valvular heart diseases, diabetes mellitus and elderly age. The above mentioned risk factors contribute to structural changes within the atrium, including its dilatation and fibrotic processes [8]. Moreover, a cardiac rhythm disturbance is a result of atrial remodelling, including structural and electrical transformation, and favouring making the changes permanent [9,10]. Making the changes in atrial structure and electric function permanent, leading to more frequent recurrences of arrhythmia episodes is more pronounced in older patients [11,12]. Besides the mentioned changes, of importance are also neurohormonal and biochemical remodelling, autonomic changes secondary to ageing process or progression of disorders of the basic cardiac functions, and also the complex of environmental and genetic factors [4]. At an initial stage of heart atrial remodelling, changes develop at the electrical level, and are characterised by a shortening of the refractory period caused by changes in L-type Ca^2+^ current passage [13]. Further changes occur at structural level, leading to hyperthrophy and fibrosis and they are a response to inflammatory process, oxidative stress, ageing and apoptosis [14].

The mechanisms leading to cardiac fibrosis are multidirectional and they involve cardiac fibroblasts, myoblasts and matrix metalloproteinases (MMPs) and their tissue inhibitors (TIMPs). The main role in the formation of extracellular matrix is played by cardiac fibroblasts accounting even for 60% of cardiac cells, exceeding the number of cardiomyocytes [1]. Cardiac fibroblasts, in principle, are inexcitable cells but they can conduct current between cardiomyocytes, what may result in non-homogeneity of the conduction of current, shortening of the functional potentials, depolarization of resting cardiomyocytes and induction of the spontaneous 4th phase of depolarization [15].

Adaptive changes of the left atrium (LA) myocytes in response to the effects of external factors are necessary for maintaining homeostasis and they lead to left atrial remodelling [10]. Left atrial fibrosis, which contributes to the development and progression of atrial fibrillation, is regarded as an important marker of the risk of adverse effects in AF patients [16]. A close interrelation is present between AF and LA remodelling. These mechanisms intertwine with each other [1]. LA dilatation favours the mechanisms predisposing to the development and persistence of arrhythmia and to LA function impairment, which is called atrial cardiomyopathy [17].

In addition to the mechanisms that are contributing to the development of AF underlying processes of left atrium fibrosis and remodelling, supraventricular beats from pulmonary veins ostia are also crucial in the initiation of AF [18,19]. Understanding the mechanisms of cardiac arrhythmias has led to the development of ablation therapy of AF. The aim of catheter ablation is to eliminate the AF triggers by modification of factors responsible for initiation and maintaining triggers. Catheter ablation of ectopic foci, that trigger arrhythmias, prevents their recurrences and reduce the incidence of chronic AF [18].

In this review paper we focused on the mechanisms of fibrosis and structural transformation of the heart atria, including neurohormonal disorders mainly of the renin-angiotensin-aldosterone system (RAAS) and activity of the profibrotic pathways initiated by the transforming growth factor beta (TGF-β), platelet-derived growth factor (PDGF) and also connective tissue growth factor (CTGF). It is worth to mention that RAAS plays the key role in the development of the syndrome of tissue responses at both electrical and structural levels [10]. We also discuss the disturbances of extracellular matrix homeostasis and the role of matrix metalloproteins and their inhibitors in fibrotic processes. An integral part of the paper is presentation of the latest data on the interactions and relationship between the above mentioned pathological processes and expression of miRNA molecules.

## 2. The Role of Inflammatory Condition in AF Induction

Since the time of the first report by Bruins et al. [20], a growing body of evidence suggest a close relationship between inflammatory condition and AF development [21,22,23]. AF frequently develops in patients with overt inflammatory conditions of cardiac (myocarditis, pericarditis) and non-cardiac origin (pneumonia and inflammatory bowel disease). Subclinical inflammatory conditions (e.g., in ischaemic heart disease) also contribute to cardiac arrhythmia development [24]. Irrespective of whether AF is the cause or the consequence of an inflammatory process, it is significantly related to oxidative stress fixed by infiltrations of the myocardium with inflammatory cells (e.g., macrophages), which is accompanied by releasing of reactive oxygen species (ROS) [25]. The inflammatory condition leads to RAAS activation and then to activation of NADPH oxidase. In consequence, these processes trigger TGF-β pathway signalling and structural and electrical remodelling of the myocardium [26]. An increased expression then occurs of various inflammatory cytokines and chemokines such as: interleukin-1 and -6, tumour necrosis factor α (TNF-α) or monocyte chemoattractant protein 1 (MCP-1) and, in consequence, a progression of chronic atrial fibrillation and AF recurrences after cardioversion are observed [24]. Inflammation plays a particular role in postoperative AF (e.g., after aortocoronary bypass grafting (CABG) or valve exchange) and catheter ablation [27,28]. In the recently conducted meta-analysis by Li T et al. [29], in 925 patients after CABG, the concentration of serum C-reactive protein was found to be a strong predictor of AF episodes. Similarly, a meta-analysis of seven studies in patients after ablation confirmed the predictive value of C-reactive protein in recurrent AF [30].

## 3. Neurohormonal Mechanisms

### 3.1. The Renin-Angiotensin-Aldosterone System (RAAS)

**Figure 1:**
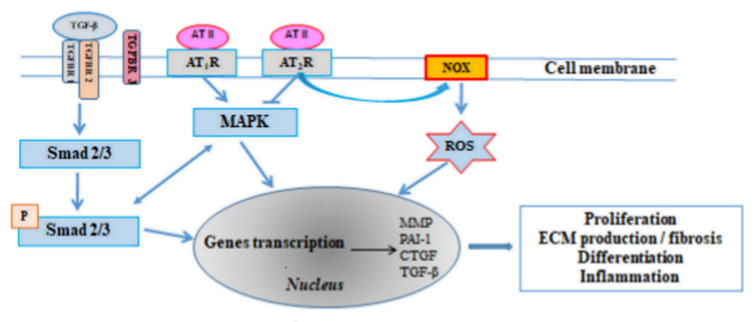
TGF-β signalling pathway and Ang II effects via AT_1_-R and AT_2_-R receptors. Ang II—angiotensin II; AT_1_R—angiotensin type I receptor; AT_2_R—angiotensin type II receptor; CTGF—connective tissue growth factor; ECM—extracellular matrix; MAPK—mitogen-activated protein kinase; MMP—matrix metalloproteinase; NOX—nicotinamide adenine dinucleotide phosphate oxidase; PAI-1—plasminogen activator inhibitor 1; ROS—reactive oxygen species; Smad 2/3—Smad family member 2/3; TGF-β—transforming growth factor β; TGFβR1,-2,-3—transforming growth factor receptor β1,-2,-3.

The renin-angiotensin-aldosterone system (RAAS) is one of the most important hormonal mechanisms controlling haemodynamic stability through regulation of blood pressure, fluid volume and sodium-potassium balance. A majority of the known proliferative, profibrotic and proinflammatory effects of angiotensin II (Ang II, AT II) occur through interaction with the type 1 angiotensin receptor (AT_1_-R). Ang II binding to AT_1_-R stimulates the mitogen-activated protein kinase (MAPK) and thus regulates the transcription of the target genes: MMP, plasminogen activator inhibitor 1 (PAI-1), CTGF, TGF-β [10] (Figure 1). The effects of activation of that pathway include fibroblast proliferation and hypertrophy, which lead to increased numbers of fibroblasts and myofibroblasts producing ECM proteins. Moreover, an increased expression of Ang II receptor was found in the left atrium in AF patients [31]. Furthermore, Goette et al. [32] demonstrated increased expressions of angiotensin-converting enzyme (ACE) and extracellular signal-regulated kinase (ERK1/2) in AF patients, which further stimulated the proliferation and activation of fibroblasts. That conclusion was confirmed in the studies demonstrating that ACE overexpression in mice led to increased atrial dilatation and fibrillation [33].

AT_1_-R stimulation leads to NADPH oxidase (NOX) activation, which is associated with intensified production of ROS, which are also involved in the expression mechanism of the above mentioned genes. Irrespective of the presented mechanism, AT_1_-R activation leads to a vasospasm mediated by inositol triphosphate (IP3) and calcium ions (Ca^2+^) and also diacylglycerol (DAG) and protein kinase C (PKC). The latter effects are, under physiological conditions, counterbalanced by Ang II interaction with type 2 receptor (AT_2_-R), leading to nitric oxide synthase (NOS) stimulation and increased NO availability, which results in relaxation of vascular smooth muscle cells [34].

Aldosterone is another RAAS effector molecule, the synthesis and release of which are stimulated by Ang II via AT_1_-R in the adrenal cortex. Through particular effects on the distal nephrons, aldosterone promotes sodium absorption, water reabsorption and potassium and magnesium loss, modulating thus the extracellular space volume and blood pressure [35,36].

High aldosterone levels are associated with myocardial hypertrophy and remodelling, proarrhythmic effect, myocardial ischaemia, coronary blood flow reduction and cardiac fibrosis. They lead to non-adaptive myocardial remodelling [37,38]. In such circumstances not only a promotion of fibrosis but also increased apoptosis, inflammation and intensification of oxidative signalling occur [39,40]. Ang II can also increase ROS production and cause cardiac hypertrophy. The molecular pathways participating in these harmful effects concern small Rho G proteins (e.g., RhoA, Rac1) [41]. These proteins act as a molecular switch, reacting with lower grade targets. Both Rho-α and Rho-β can be regulated by Ang II via AT_2_-R effect, and the Rho kinases are associated with ROS promotion and vasculitis mediated by direct NO synthase activation in the vascular endothelium [42]. On the other hand, Rac1 is associated with time- and aldosterone dose-dependent increase of peroxide production, which is an effect abolished by eplerenone [43]. In the cardiomyocytes [44], cardiac troponins are phosphorylated by Rho kinases in order to prevent contractile apparatus tension. On the other hand, Rho kinase inhibition by Fasudil prevents the development of cardiac hypertrophy and diastolic heart failure [41].

RAAS activation in chronic heart failure (CHF) and AF leads to pathological consequences associated with hypotension and hypoperfusion caused by CHF and/or AF. They include retention of salt and water by the kidneys, blood vessel narrowing and structural remodelling and also fibrosis of the cardiac atria [45]. Initially, these changes lead to blood pressure elevation and thus to changes of tissue perfusion. In the long run however, they lead to pressure increase in the left atrium and to its dilatation as well as to myocardial fibrosis and remodelling. That creates a dangerous positive feedback loop, exacerbating in CHF and predisposing to AF, and thus a further RAAS activation.

### 3.2. Transforming Growth Factor β (TGF-β)

Transforming growth factor β (TGF-β) has five isoforms, three of which have been found in humans (TGF-β1, TGF-β2, TGF-β3). TGF-β is one of the strongest stimulators of collagen synthesis by cardiac fibroblasts [10]. It exerts its effect through binding to specific receptors expressed on all cell types. Three types of the receptors (TβR-I, -II, -III) have been found in the extracellular space as yet. Binding of a ligand to the receptor causes a phosphorylation reaction cascade, in which the inactive Smad 2, 3 proteins form a Smad complex [46]. The Smad complex moves then into the cell nucleus, where it binds to appropriate regulation regions and induces the expression of genes involved in the fibrosis process [47], what leads to production of a matricellular protein with a profibrotic effect, which is released into the ECM. That protein modulates intercellular interactions and additionally stimulates the synthesis of ECM proteins. However, it is not directly involved in the structure and organization of ECM [48].

Besides fibroblast activation and collagen synthesis, TGF-β can also induce cardiomyocyte apoptosis [49]. It should be also stressed that angiotensin II cannot cause cardiac hypertrophy and fibrosis in absence of TGF-β, but can induce TGF-β synthesis, Smad 2/3 phosphorylation and Smad complex translocation into the cell nucleus and can increase active Smad 2/3 binding to DNA (Figure 1). TGF-β can also directly stimulate the expression of type 1 angiotensin II receptor [50]. Angiotensin II predisposes also to fibrosis through promoting of expression of profibrotic factors such as endothelin-1. Moreover, angiotensin II, combined with aldosterone, promotes oxidative stress (i.e., excessive ROS production) and inflammatory condition, mainly through NADPH oxidase activation [51,52].

Oxidative stress and increased production of ROS are also involved in TGF-β activation and production, which confirms that they are an important mediators in the fibrotic process and are involved in the initiation and maintenance of AF [53].

TGF-β expression is also regulated by hypoxia-inducible factor (HIF-1α) that is also involved in the pathogenesis of atrial fibrosis process and AF development. Su et al. [54] showed positive correlation between the expression of HIF-1α and the extent of myocardial fibrosis, indicating that HIF-1α can promote the expression of TGF-β and thus induce atrial fibrosis.

### 3.3. Platelet-Derived Growth Factor (PDGF)

Platelet-derived growth factor (PDGF) is also involved in the regulation of collagen synthesis and, more generally, proteins included in the ECM. Several isoforms of that factor are present: PDGF-AA, -AB, -BB, -CC and -DD [46,55], all with similar properties but different signalling pathways. The extensive study by Zhao et al. [56] revealed that PDGF-DD significantly increased the proliferation of cardiac fibroblasts, their differentiation to myofibroblasts (myoFb) and synthesis of collagen type I. Moreover, significantly higher levels were found of matrix metalloproteinases: MMP-1, MMP-2 and MMP-9 in PDGF-DD treated cells, which were in agreement with the increased expression of their inhibitors: TIMP-1 and TIMP-2. The increase of both TIMPs and MMPs is a counterbalance to collagen degradation. The profibrogenic effect of PDGF-DD is mediated by TGF-β pathway activation. These findings show that PDGF-DD promotes fibrogenesis through many mechanisms [56]. PDGF-AA, -BB and to some extent -CC also are able to cause cardiac fibrosis as demonstrated in the model of transgenic mice [57]. Their action possibly includes activation and participation of pathways similar to PDGF-DD.

It should be noted, that in the regulation of PDGF profibrogenic activity are also involved hypoxia and HIF-1α [58]. It has been established that HIF-1α levels were increased in patients with AF suggesting that hypoxia process plays an important role in the structural remodeling and pathogenesis of AF.

### 3.4. Connective Tissue Growth Factor (CTGF)

Connective tissue growth factor (CTGF) is a member of a small family of proteins, which are characterised by highly conserved disulfide bond pattern and have 3–4 homologous domains with relevant proteins [59,60]. They include: domain 1—homologous with proteins binding the insulin-like growth factor (IGF-1), domain 2—homologous with type C von Willebrand factor, domain 3—homologous with thrombospondin type 1 and domain 4—containing cysteine knot motif, which is common to proteins that bind to heparan sulphate proteoglycans (HSPGs) [61].

CTGF released from cells shows an ability of interaction with many different molecules (e.g., proinflammatory cytokines, growth factors or cell surface receptors). It is believed that interactions of CTGF with various molecules lead to positive or negative changes in signal transduction pathways. Furthermore, through modulations of signal transduction, it causes changes in adhesion and migration of cells, in angiogenesis process, vascular permeability, differentiation including formation and activation of myofibroblasts and, in effect, remodelling of the extracellular matrix leading to tissue remodelling and changes in organ structure [61].

CTGF, modulating numerous cellular processes plays the central role in the pathogenesis of many diseases, in which tissue remodelling occurs. CTGF expression is induced by many cytokines, factors exerting stimulating or inhibiting effects, external stimuli the participation of which is associated with development of diseases of various origin [61]. The presence of CTGF induces formation of myofibroblasts through transdifferentiation of other cells, including epithelial cells [62], residential fibroblasts [63] or fibrocytes, which have been recruited into an organ through mediation of chemokines [64]. CTGF also activates myofibroblasts and stimulates their deposition and also remodelling of ECM proteins. These processes lead to tissue remodelling and fibrosis. Tissue remodelling occurring in blood vessels may be a cause of local arterial hypertension development, which can intensify CTGF expression [65] creating thus a positive feedback loop leading to greater tissue remodelling. CTGF induces also expression of various cytokines, such as TGF-β [66] and VEGF [67], which further deepen CTGF expression. The presented relationship demonstrates many positive feedback loops including CTGF expression, which, working together, can contribute to further progression of the fibrotic process. CTGF inhibition can cause a blockade of these positive feedback loops, making possible restoration of the normal structure and function of the organs.

**Figure 2:**
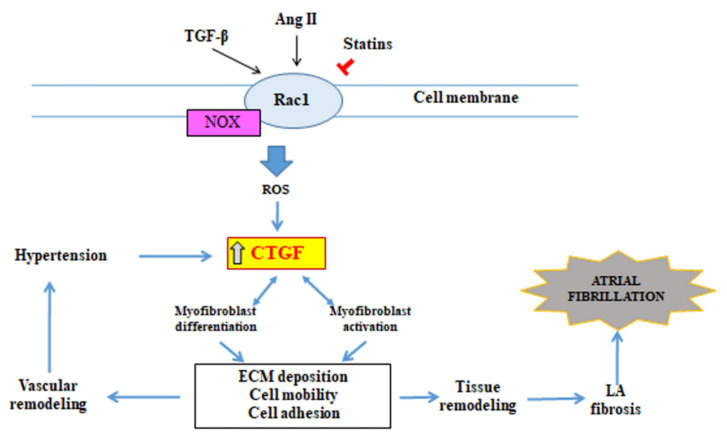
CTGF as the central mediator of tissue remodelling and atrial fibrosis. ⟙ (red arrow) inhibition; LA—left atrium; Rac 1—Rac family small GTPase 1.

CTGF is the basic modulator of many signalling pathways involved in the atrial fibrotic process, in the first place including Ang II- and TGF-β-dependent pathways [68]. Ang II stimulates CTGF through activation of the G—Rac 1 protein [69]. The small GTP-binding protein—Rac 1 is a member of the Rho superfamily of GTPases, intracellular signal transmitters, which participate in the regulation of NADPH-dependent oxidative stress [70]. The Rac1 activity in NADPH-dependent production of reactive oxygen species is increased in the atria both in patients and animals with AF [71,72,73]. It is very likely that this Rac 1 activity underlies the pathogenesis of AF (Figure 2).

In view of the fact that Rac 1 requires modification after translation through isoprenylation for normal functioning, it can be potentially inhibited by 3-hydroxy-3-methyl-glutaryl-coenzyme A reductase inhibitors, which block the synthesis of isoprenoids [74]. Indeed, statins inhibit the angiotensin II-induced, NADPH oxidase-dependent myocardial oxidative stress and heart remodelling [74,75]. Furthermore, the treatment with statins is associated with a reduced incidence of AF development in postoperative patients [75].

## 4. Participation of Extracellular Matrix, Matrix Metalloproteinases and Their Inhibitors in Fibrotic Process

Matrix metalloproteinases belong to the family of zinc-dependent proteolytic enzymes, which participate and, at the same time, regulate extracellular matrix turnover. Tissue metalloproteinase inhibitors, being in equilibrium with metalloproteinases, take part in that process [6,76]. The key physiological role of MMP in the body is degradation of the proteins contained in the ECM and also in the basement membrane through disruption of collagen networks and recruiting of proinflammatory cells [77]. The extracellular matrix proteins include mainly structural ECM proteins: elastin, collagen, fibronectin, laminin, proteoglycans, glycoproteins. Important ECM elements also include regulator proteins, such as MMP and their inhibitors [78,79].

ECM homeostasis maintains the balance between synthesis and degradation processes. That balance may be disrupted in favour of ECM protein synthesis processes in the presence of profibrotic stimuli, such as proinflammatory cytokines or intensification of oxidative stress, contributing thus to excessive fibrosis. ECM homeostasis disruption underlies the pathogenesis of AF [80,81]. Many reports confirm that ECM degradation by MMP and thus intensification of fibrotic processes underlies also the pathogenesis of other cardiovascular diseases, i.e., atherosclerosis, restenosis, dilated cardiomyopathy or myocardial infarction [82]. Experimental clinical studies [83] have confirmed the importance of fibrosis in the cardiac atria in patients with AF. Such spectacular fibrotic lesions have not been found, however, in the cardiac ventricles of AF patients. Ventricular fibrosis in such patients frequently leads to an impairment of the adequate contractility and relaxation of the heart and, in the future—to HF. Heart fibrosis processes can simultaneously occur in AF and in HF. Both these conditions can intertwine with each other, being simultaneously their cause and consequence. If these diseases coexist, they significantly impair the prognosis for the patients, increasing the risk of death related to heart failure exacerbation [83].

The regulation of expression of metalloproteinases occurs at various levels. The transcription process can be regulated by various cytokines, e.g., TNFα. TNFα both induces MMP transcription and activates MMP through activation of various proteases by paracrine pathway [84]. MMPs are secreted as inactive forms (zymogens) requiring activation [82]. MMP activation can be then blocked by TIMPs [82]. A shift of the balance towards MMPs results in an increased proteolysis of ECM proteins, while a shift towards TIMPs causes an opposite effect, i.e., protects the proteins against excessive degradation [85]. Pathological processes damaging the heart lead to an increased activity of metalloproteinases with simultaneous reduction of the levels of their tissue inhibitors.

A well-controlled balance between MMPs and TIMPs ensures maintaining of ECM homeostasis [78,79]. Under normal conditions, i.e., health, that balance between MMPs and TIMPs is controlling much more than just ECM degradation. TIMPs can directly inhibit ECM degradation but also can indirectly regulate the ECM turnover, while the definite effect ascribed to the inhibitors depends on the type of metalloproteinase inhibited by TIMP and also on the environmental conditions (state of health vs. disease). Increased TIMP levels lead to ECM deposition or fibrotic processes, while TIMP loss causes a prolonged ECM degradation [85]. Thus, a lack of balance between MMPs and TIMPs translates into the extent of pathological remodelling of myocardial ECM with intense fibrotic processes, which frequently take place in cardiac atrial remodelling [82,86]. It is also worth to pay attention to the fact that MMPs, as well as TIMPs regulate the degradation of collagen and other ECM proteins in the heart atria. That may be exemplified by MMP-1, which degrades collagen type I, II and III; MMP-2 and MMP-9, degrading collagen type I, III, IV, V and VI; MMP-12, degrading elastin and MMP-8 and MMP-13, degrading collagen type I and III [77]. In a healthy heart, collagen deposition is limited to maintaining the cardiac architecture. However, with progression of various heart diseases, the combined collagen network undergoes quantitative and qualitative changes, leading to excessive accumulation of collagen in the regions of cardiomyocyte loss (e.g., in myocardial infarction, repair fibrosis) or accumulation of dispersed collagen in the myocardium (e.g., in dilated cardiomyopathy) [87].

Both metalloproteinases and tissue inhibitors of metalloproteinases can play the role of biomarkers. Their levels in the circulation are of prognostic value and enable to estimate the mortality rate due to heart failure. This is particularly true of the level of TIMP-1, which is a specific MMP-9 inhibitor. The stage of heart failure is correlated with the level of metalloproteinase inhibitors. Therefore, MMP activity increases together with TGF-β1 factor expression in the myocardium, promoting fibrosis, and it correlates with the intensity of the inflammatory condition and with that of oxidative stress [88]. In summary, it seems that the use of TIMPs but also MMPs as biomarkers may be also a therapeutic target in patients with diseases associated with uncontrolled degradation of the extracellular matrix proteins and thus it could provide valuable information about the current condition of the heart [89].

The potential role of fibrosis and ECM remodelling in atrial fibrillation has been supported by animal studies and clinical cross-sectional studies. It is also worth to pay attention to the prospective study, Atherosclerosis Risk in Communities (ARIC) [90], which suggests that increased MMP levels are associated with a higher risk of ischaemic heart disease. In contrast to that, another study [91] only illustrates the associations between MMPs and TIMPs and the inflammatory conditions and shows no direct relation to the coronary risk [91].

In response to the proinflammatory and profibrotic factors, or increased synthesis of ECM proteins (mainly collagen I, III and VI) an activation of cardiac fibroblasts and transdifferentiation into myofibroblast phenotype occur, effectively stimulating connective tissue synthesis. That explains the progression of fibrosis then, when MMP activity is high, in spite of the fact that main MMP function is degradation of extracellular matrix proteins [92,93]. Myofibroblasts are characterised by a changed morphology, increased ability to synthesize ECM proteins and MMPs [80] and a twice higher ability to synthesize collagen compared with cardiac fibroblasts [94]. A strong collagen synthesis stimulator is the TGF-β factor, which also participates in cardiac fibrosis process, contributing to differentiation of the fibroblasts into myofibroblasts, exerting its effect via the Smad signalling pathways [95,96]. In the experimental studies in murine model with increased and prolonged TGF-β expression, the fibrosis was confirmed in the atria but not in the ventricles [97]. Therefore, the effect of myofibroblasts is particularly targeted at fibrotic process promoting [98]. An intensified fibrosis contributes to hypertrophy of the cardiomyocytes or to their loss. Moreover, the fibrosis process, inflammatory condition and ECM degradation, mediated by myofibroblasts and macrophages, respectively, lead to an impairment of electrical conduction by cardiomyocytes and proteins of the extracellular matrix. Cardiac fibroblasts participate in the electrical remodelling in AF, in view of their electrophysiological properties compared with surrounding cardiomyocytes. That results in non-homogeneity of the conduction of current, shortening of the functional potentials, depolarisation of resting cardiomyocytes and spontaneous induction of phase 4 depolarisation, which has been already mentioned in Section 1 [15]. That unfavourable environment, besides impairing electrical conduction in the heart, leads to a loss of active cardiomyocytes, which is observed in patients with AF [80]. To summarize, fibrosis impairs electrical conduction between cardiomyocytes, which is caused by intense fibrogenesis and increase of extracellular volume (ECV) of proteins. It has been found that both the composition and volume of the ECM are closely correlated with AF [99].

It should be also stressed that many, frequently simultaneously occurring events underlie the pathogenesis of atrial fibrillation, including the pathways of synthesis and degradation of extracellular matrix proteins and numerous metalloproteinases and their inhibitors involved in these pathways. A deeper understanding of the complexity of the mechanisms regulating the interrelationship will provide a detailed view into the pathogenesis of AF and may establish new potential therapeutic goals.

## 5. The Participation of MicroRNA in the Regulation of Signalling Pathway Involved in the Pathogenesis of Atrial Fibrillation

MicroRNA (miRNA, miR) is a class of single-stranded, short (~22-nucleotide), evolutionarily-conserved, non-coding RNA. The molecules of miRNA participate in post-transcriptional regulation of gene expression through protein translation blocking or degradation of the target mRNA [100]. Single miRNA can regulate the expression of many genes, while a single mRNA can be regulated by one or several types of miRNA. Over 1/3 of protein-encoding genes in human cells are regulated by miRNA [101,102]. miRNA regulates the expression of genes involved in many processes, both physiological and pathological: cell proliferation and differentiation, regeneration, ageing, apoptosis, angiogenesis, oncogenesis [100,103]. Much data is available suggesting miRNA participation in both normal heart development and in the pathophysiology of many cardiovascular system diseases including: coronary artery disease, myocardial infarction, heart failure or atrial fibrillation [104,105,106,107]. miRNA molecules can be released by the cells into the bloodstream, where they circulate in the form of stable complexes with proteins or in microvesicles [108]. Much data is available stressing the important role of circulating miRNA molecules and suggesting their application as potential biomarkers of diagnostic, prognostic and also predictive value in the pathogenesis of cardiovascular system diseases [109,110].

**Figure 3:**
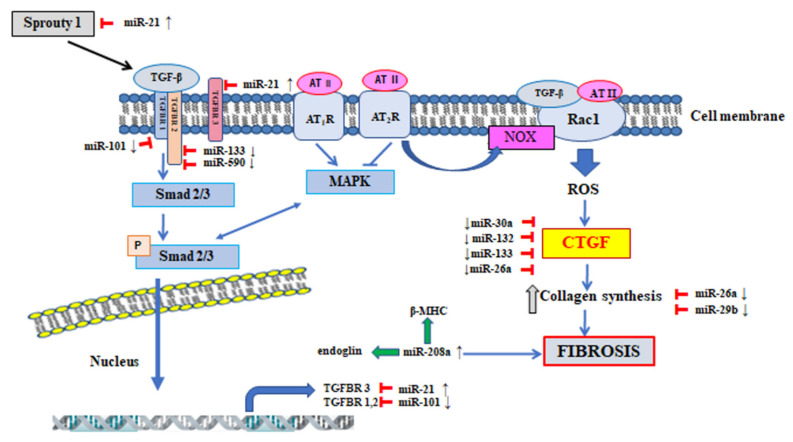
Modulation of signalling pathways by miRNAs in the pathogenesis of atrial fibrosis. ↑ up regulation of miR; ↓ down regulation of miR; ⟙ (red arrow) inhibition; **→** (green arrow) activation. miR—microRNA; β-MHC—β-myosin heavy chain; Sprouty 1—protein Sprouty homolog 1.

In the course of atrial fibrillation, changes in the miRNA profile occur, both in the heart atrial tissue and in the bloodstream [111,112,113]. The participation has been studied of specific miRNA molecules in the regulation of individual processes underlying AF development, with particular consideration of atrial remodelling at structural level, during which connective tissue deposition and intense fibrosis are seen [113,114]. Many molecules and signalling pathways are involved in the myocardial fibrosis process, the expression or activities of which are subjected to a positive or negative regulation by miRNA, creating a network of interrelations [115,116]. The regulation of profibrotic mediators, important in the process of atrial fibrosis, i.e., TGF-β, CTGF, MAPK signalling pathway, Ang II, IGF-1 [3,115,116], involves various miRNA molecules directly associated with atrial fibrosis in AF, that is: miR-21, miR-26a, miR-29b, miR-30a, miR-133, miR-101, miR-132, miR-208a/b [3,4,113,117] (Figure 3).

Some miRNA types can enhance the fibrotic process through direct intensification of expression or activity of TGF-β-dependent signalling pathway molecules. Some papers [118] revealed that increased miR-21 expression in fibroblasts was associated with atrial fibrotic process. The mechanism of action of miR-21 takes two forms. The first one includes inhibition of the activity of the gene encoding TβR-III receptor, which is a negative regulator of the TGF-β-Smad 3 signalling pathway (Figure 3) [119]. The second mechanism of miR-21 action includes inhibition of expression of the gene encoding protein Sprouty homolog 1 (Spry1). The protein Sprouty 1 participates in the control of TGF-β factor releasing and inhibits the activity of the extracellular signal-regulated kinases signalling pathway and mitogen-activated protein kinase signalling pathway (ERK-MAPK) [120]. In the rat experimental model induced by ischaemic heart disease and in patients with AF it was demonstrated that increased miR-21 expression was associated with a reduction of Sprouty 1 protein level in atrial tissues [118]. miR-21 can also intensify the process of atrial fibrosis through modulation of inflammatory processes mediated by phosphorylation of the transcription factor signal transducer and activator of transcription 3 (STAT3) [121]. The experimental studies [121] conducted in rats with pericarditis and AF demonstrated that miR-21 expression inhibition through administration of antagomir-21 suppressed the process of STAT3 protein phosphorylation, inhibited expression of the genes encoding proteins associated with the atrial fibrosis process and reduced the risk of AF development.

A profibrotic action is also shown by miR-208a, which intensifies the expression of endoglin-encoding genes, and β-myosin heavy chain (β-MHC) [122]. Cañón et al. [123] noted an increased expression level of miR-208a and miR-208b in the cardiac tissue of patients with AF. An analysis of the predictions of the target genes has demonstrated that miR-208a and miR-208b interact directly with Sox5 and Sox6 proteins belonging to negative transcription factors of MYH7 protein. The function of Sox5 and Sox6 proteins is associated with the heart rate and some electrophysiological features. In vitro studies revealed that miR-208a and miR-208b overexpression caused suppression of the Sox5 and Sox6 proteins, respectively. The studies conducted as yet demonstrated that inhibition of miR-208 expression in various experimental models of heart failure effectively protected against fibrotic process and cardiac hypertrophy. In view of the presence of the above mentioned processes in the pathogenesis of AF, miR-208 can be considered as a potential therapeutic target [114,123].

Another molecule showing protective and antifibrotic effects is miR-101, the mechanism of action of which also includes inhibition of the expression of the proteins involved in the regulation of TGF-β signalling pathway. It was demonstrated that the target genes for miR-101 included gene encoding protein TβR-I (Table 1) [124]. In the study by Lu et al. [125], conducted both in a canine experimental model and in patients with AF, a decreased level of miR-101 expression was noted in the atrial tissue. It can be therefore speculated that the reduced miR-101 expression level is associated with an intensification of atrial fibrosis process in the course of AF. On the other hand, an experimental increase of miR-101 expression could bring about a beneficial effect in the form of reduction of unfavourable atrial remodelling.

Another important pathway involved in the process of atrial fibrosis is the CTGF-dependent pathway, while the expression of CTGF signalling molecule is regulated again by TGF-β and endothelin. In the effect of CTGF action, collagen synthesis is increased. In the regulation of CTGF expression four miRNA molecules are involved: miR-30a, miR-133, miR-26a, miR-132 (Table 1) [117,126,129]. A direct inhibition of CTGF expression by miR-30a and miR-133 causes an alleviation of the fibrotic process. In the experimental studies conducted in rabbit model, in the atrial tissue a decreased miR-30a expression level and increased levels of Snail 1 transcription factor and Periostin were detected and intensified fibrosis were found [127]. The role of miR-30a in the regulation of the Snail 1 and Periostin proteins was studied through both overexpression and inhibition of miR-30a in rat cardiac fibroblasts [127]. In the case of AF patients, a decreased miR-30a expression level was found in the atrial tissue [131]. The functional studies concerning miR-133 and miR-590 revealed other target genes than in the case of miR-30a, and they included TGF-β and TβR-II. The studies conducted in canine experimental model demonstrated that miR-133 and miR-590 transfection into fibroblasts in the cardiac atria reduced the levels of TGF-β and TβR-II and the content of collagen. It is worth to mention that this phenomenon was reversible after anti-miR administration [128].

The antifibrotic mechanism of action of the next molecule, namely miR-26a includes a direct inhibition of CTGF expression and interaction with the gene encoding collagen type 1 (COL1) (Table 1) [129]. In the experimental studies conducted in dogs with heart failure accompanied by AF, a reduction of miR-26a expression in the left atrium was found, which corresponded to an increased expression level of the gene encoding the Ca^2+^-permeable transient receptor potential canonical-3 (TRPC3) protein. The increased TRPC3 protein level was associated with intense activation, proliferation and differentiation of atrial fibroblasts [132].

The studies by Qiao et al. [117] also presented an antifibrotic mechanism of miR-132 action, which resulted from negative regulation of CTGF protein expression (Table 1). It was demonstrated that Ang II administration into cardiac fibroblasts was associated with an increased miR-132 and CTGF expression, what confirmed the key role of these molecules in the fibrosis process. In further studies the authors observed, both in humans and in an animal model, a reduced miR-132 expression and increased CTGF protein expression level in the process of structural atrial remodelling in the course of AF. The experimental miR-132 inhibitor administration was associated with an intense fibrosis process, while cardiac fibroblasts transfection with miR-132 mimic led to an alleviation of that process in view of CTGF protein level reduction. The authors postulate that the mechanism of miR-132 action, associated with fibrotic process alleviation, can find a potential therapeutic use in AF [117].

Another molecule regulating the expression of genes encoding proteins participating in collagen biosynthesis and demonstrating an antifibrotic effect is miR-29b (Table 1). The target genes for miR-29b include COL1α1, COL1α2, COL3α1, elastin and fibronectin. A reduced miR-29b expression was noted in the cardiac atrial tissue and in fibroblasts sampled from dogs with induced ventricular tachycardia. An experimental increase of miR-29b expression was associated with an expression reduction of the genes encoding proteins responsible for synthesis of collagen type I and type III (COL1α1, COL3α1) and with a reduction of ECM remodelling, and conversely [130].

The knowledge of the functions fulfilled by individual miRNA molecules in the process of atrial fibrosis enables a deeper insight into the molecular background of AF. miRNA molecules can play the function of both activators and inhibitors of the secreted profibrotic factors. Keeping the balance between miRNA molecules demonstrating anti- and profibrotic effects is of key importance for controlling the atrial fibrosis process.

The presented miRNA molecules (Table 1), showing both pro- and antifibrotic effects, participate in the regulation of the expression of genes encoding the most important proteins of the signalling pathways involved in the processes of atrial fibrosis [4,113]. The driving force of fibrosis processes is the fact that in all experimental models analysed, an increase of miRNA expression of profibrotic character and a decrease of miRNA expression of antifibrotic character occur. Furthermore, miRNA-21 and miR-208a/b, through a direct increase of the expression of the genes exerting profibrotic effects, positively modulate the process of pathological atrial fibrosis. On the other hand, miR-101, miR-30a, miR-133, miR-590, miR-132, miR-26a and miR-29b, through a negative regulation of the expression of the molecules exerting profibrotic effects, inhibit the atrial fibrotic process. In the process of pathological atrial fibrosis the balance between miRNA molecules with pro- and antifibrotic effects is disturbed. In the case of miRNA molecules promoting the fibrotic process, an increased expression level can be expected while the level of antifibrotic miRNA expression is decreased, which is also associated with a derepression of the profibrotic target genes. Both miRNA groups (pro- and antifibrotic) are important therapeutic targets. Such targets would include a repair of the disturbed expression level of a given miRNA, which could reverse the process of pathological fibrosis. An experimental increase of the expression of miRNA molecules exerting antifibrotic effects and a reduction of the expression of miRNA types promoting fibrosis (profibrotic action) could produce a protective effect against fibrosis.

## 6. Conclusions and Perspectives

Atrial fibrosis should be regarded as a potential key factor and biomarker of AF pathogenesis. Fibrotic tissue formation is a complex multifactorial process involving many interactions of neurohumoral and cellular factors. The studies presented in the paper seem to clearly suggest that the Ang II-MAPK and TGF-β-Smad signalling pathways and Rac1-dependent CTGF activation play an important, even crucial role in the direct or indirect regulation of atrial remodelling and fibrosis in AF. An extremely important role in the regulation of the fibrotic process and ECM remodelling is also played by miRNA molecules. The miRNA molecules exert both pro- and antifibrotic effects. They participate in the regulation of the expression of genes encoding the most important proteins of the signalling pathways involved in fibrosis. Keeping of the balance between miRNA molecules exerting anti- and profibrotic effects is of the key importance for the control of atrial fibrosis in AF (Table 1).

Inflammatory status and oxidative stress are important elements contributing to changes in the ECM. The ECM metabolism is a process regulated in a strict and dynamic way in the cardiac tissues by the balance of the degrading enzymes and their endogenous inhibitors. The co-operation between MMPs and TIMPs is regulated by growth factors, inflammatory cytokines and ROS. An understanding of the molecular mechanisms mediating the MMP/TIMP balance may contribute to a new look at remodelling of the heart atria and to development of effective drugs which could prevent AF or reverse its pathogenesis.

**Figure 4:**
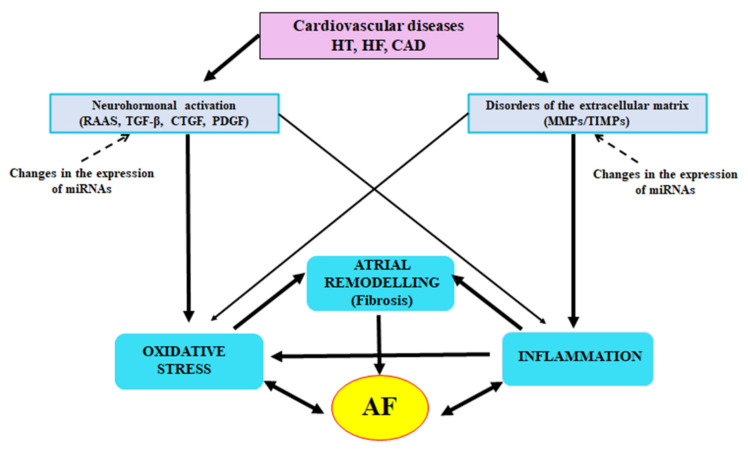
Possible association of atrial fibrillation with atrial remodelling, inflammation and oxidative stress. ⤏ modulation of the expression; → activation of the expression. AF—atrial fibrillation; CAD—coronary artery disease; HF—heart failure; HT—hypertension; PDGF—platelet-derived growth factor; RAAS—renin-angiotensin-aldosterone system; TIMP—tissue inhibitor of metalloproteinase.

The cellular and molecular control of atrial fibrosis is very complex (Figure 4). The recent years have brought much new information increasing our knowledge of AF pathomechanisms, but there is still much to investigate. The greatest challenge will be using the knowledge of these mechanisms in the search of new biomarkers in order to determine the extent of fibrosis and to monitor the therapy, and also in the search of novel therapeutic methods changing the natural history of AF through prevention of fibrosis development.

## Figures and Tables

**Table 1:** miRNA molecules involved in the regulation of the signalling pathways in the process of atrial fibrosis.

miRNA	Effect	Regulation in Fibrosis	Signal Pathway	Target Genes	References
miR-21	profibrotic	up	TGF-β-Smad 3 ERK-MAPK	TβR-III	[118,119,120,121]
				Spry1	
				STAT3	
miR-208a/b	profibrotic	up	TGF-β	Endoglin	[115,122,123]
				β-MHC	
				Sox5	
				Sox6	
				THRAP1	
miR-101	antifibrotic	down	TGF-β	TβR-I	[124,125]
miR-30a	antifibrotic	down	CTGF	CTGF	[126,127]
				Snail 1	
				Periostin	
miR-133	antifibrotic	down	CTGF	CTGF	[126,128]
				TGF-β	
				TβR-II	
miR-590	antifibrotic	down	TGF-β	TGF-β	[128]
				TβR-II	
miR-132	antifibrotic	down	CTGF	CTGF	[117]
miR-26a	antifibrotic	down	CTGF	CTGF	[129]
			PI3K-AKT	COL1	
miR-29b	antifibrotic	down	ERK	COL1α1	[130]
				COL1α2	
				COL3α1	
				Elastin	
				Fibronectin	

COL—collagen; ERK—extracellular signal-regulated kinase; ERK-MAPK—extracellular signal-regulated kinase signaling pathway and mitogen-activated protein kinase signaling pathway; PI3K-AKT—Phosphatidylinositol 3-kinase—protein kinase B; Snail 1—zinc finger protein; Spry 1—protein Sprouty homolog 1; Sox 5, -6—Transcription factors Sox 5, -6; STAT3—signal transducer and activator of transcription 3; TβR-I, II, III—transforming growth factor receptor β-I, -II, -III; THRAP1—mediator of RNA polymerase II transcription subunit 13.

## Data Availability

Not applicable.
